# Pedestrian and Animal Recognition Using Doppler Radar Signature and Deep Learning

**DOI:** 10.3390/s22093456

**Published:** 2022-05-01

**Authors:** Danny Buchman, Michail Drozdov, Tomas Krilavičius, Rytis Maskeliūnas, Robertas Damaševičius

**Affiliations:** 1 Department of Applied Informatics, Vytautas Magnus University, 44404 Kaunas, Lithuania; tomas.krilavicius@vdu.lt (T.K.); rytis.maskeliunas@vdu.lt (R.M.); robertas.damasevicius@vdu.lt (R.D.); 2 JVC Sonderus, 05200 Vilnius, Lithuania; michail.drozdov@gmail.com

**Keywords:** doppler radar, micro-Doppler signature, pedestrian recognition, animal recognition, deep learning

## Abstract

Pedestrian occurrences in images and videos must be accurately recognized in a number of applications that may improve the quality of human life. Radar can be used to identify pedestrians. When distinct portions of an object move in front of a radar, micro-Doppler signals are produced that may be utilized to identify the object. Using a deep-learning network and time–frequency analysis, we offer a method for classifying pedestrians and animals based on their micro-Doppler radar signature features. Based on these signatures, we employed a convolutional neural network (CNN) to recognize pedestrians and animals. The proposed approach was evaluated on the MAFAT Radar Challenge dataset. Encouraging results were obtained, with an AUC (Area Under Curve) value of 0.95 on the public test set and over 0.85 on the final (private) test set. The proposed DNN architecture, in contrast to more common shallow CNN architectures, is one of the first attempts to use such an approach in the domain of radar data. The use of the synthetic radar data, which greatly improved the final result, is the other novel aspect of our work.

## 1. Introduction

Artificial intelligence (AI), pattern recognition, machine learning (ML), and deep learning (DL) have recently gained popularity in a variety of domains of application, including autonomous driving [1], Internet-of-Things (IoT) [2], robots [3], smart mobility [4], etc. These applications collect data from their surroundings by sensing it; then they analyze the collected data, making choices and taking actions depending on the analysis [5]. Furthermore, computational intelligence methods and inference techniques based on deep learning are being intensively explored to improve the accuracy of computer vision systems.

One of the most significant challenges in computer vision is object tracking [6,7]. It offers a wide range of real-world applications, including robotics, medical imaging, traffic monitoring, autonomous vehicle tracking, surveillance [8], etc. Despite the obstacles of visual tracking, researchers are encouraged to develop quicker and better approaches, including resilience to strong occlusions, extreme size shift, discontinuities, precise localization, multi-object tracking, and failure recovery [9]. Despite achievements in resolving multiple obstacles under a variety of conditions, the basic issues remain complicated and difficult [10]. Because of its widespread applications in domains, such as gesture recognition [11], driver tracking [12], human action recognition [13], sports analysis [14], industrial work activity [15], monitoring the condition of industrial machinery [16], visual surveillance [17], and healthcare and rehabilitation [18], visual object tracking (VOT) is an active research issue in computer vision and machine learning. However, tracking is complicated by features such as partial or full occlusion, backdrop clutter, light change, deformation, and other environmental factors [19].

Pedestrian detection is a critical problem in many intelligent video surveillance systems because it offers critical information for semantic comprehension of video [20]. Accurate recognition of individual pedestrian occurrences in images and videos is critical in a variety of applications that might improve the quality of human existence. This pedestrian detection can be performed using radar [21,22,23,24].

Today, radar has multiple applications—from homeland security, through local radar for automotive [25], city surveillance [26], military [27] and even for healthcare [28] purposes. For example, a radar sensor can be capable of detecting the presence of a worker’s activity and highlighting movements away from the workstation [29]. Target categorization is a critical radar activity in a wide range of security and military applications. Some applications, such as video surveillance [30], make use of electro-optical (EO) sensors. Radar offers substantial advantages over EO sensors in terms of resistance to harsh weather and poor illumination conditions, low cost, and durability, according to [31].

The micro-Doppler signature created by a subject reacting to an active emitter, for example, a radar, laser, or even acoustic emitters can be used to monitor the subject’s minuscule micromotions, or even only sections of a subject. The micro-Doppler signatures are generated by the kinematic parameters of the object’s motion and may be used to acquire the prominent elements of the object’s motion and, in certain cases, to identify the object [32]. The target classification using the radar data traditionally uses one or several of the approaches highlighted in [33]:

1. Classification based on target radar cross-section (RCS) estimates.
2. Classification based on target RCS ratios.
3. Classification based on target RCS distributions.
4. Classification based on target modulation signatures.
5. Classification based on the target polarization scattering matrix.
6. Classification based on other scattering mechanisms.
7. Classification based on target kinematics.

Although many of these methods are more useful while dealing with man-made objects such as planes, [34], ships [35], drones [36], helicopters, [37] and other vehicles [38], there are some common problems with a classification of a walking person, an animal, a cyclist, or a group or its moving patterns [39,40]. The major problem while using any radar cross-section-based (RCS) approach is the calibration procedures of the radar. The result of RCS estimation is highly sensitive to the range of the target, the material, and the aspect angle.

For a long time, the most promising approaches to target classification were based on the decomposition of manually specified feature vectors using one or the other of several decomposition techniques [41,42,43]. The most widely used decomposition methods are principal component analysis (PCA) [42,43] and singular value decomposition (SVD) [41]. However, recently, deep-learning methods such as deep convolutional neural networks (DCNNs) have been adopted for radar-based target detection and recognition tasks [44,45,46]. DCNNs have been used to process several forms of millimeter-wave radar data [47] as well as light detection and ranging (LiDAR) data [48].

The MAFAT radar challenge [49] is a perfect opportunity to test different approaches to target classification without investing a huge effort in data acquisition, annotation, and other required steps before the data can be used for the analysis. The contributions of this study are as follows: a novel custom deep-learning architecture for solving the animal and pedestrian recognition problem, which has not been used before. To the best of our knowledge, the suggested deep neural network (DNN) architecture is one of the first attempts to employ such a method in the domain of radar data as opposed to more usual shallow convolutional neural network (CNN) designs. The other innovative component of our study is the use of synthetic radar data, which enhanced the final outcome considerably. Our competition technique might be utilized as a foundation for future implementations of radar classification based on CNN.

The paper is arranged as explained further. Section 2 analyzes state-of-the-art work. The problem of the MAFAT radar challenge, the data pipeline, and the proposed deep neural architecture are described in Section 3. The dataset used in this study and the experimental results are presented and analyzed in Section 4. Finally, the results of this study are discussed in Section 5.

## 2. Related Work

Some examples of using neural networks as the main classification module can be found as early as 1996 [50]. More recent related results include [51,52]. The common trend in this research is that very small data samples are used (tens or hundreds) and networks are very shallow (3–4 layers).

Gadde et al. [53] proposed strategies for analyzing radar data and using them to detect geriatric falls. The disparity in radar signal returns and the Doppler shift are caused by human motor activity. Because the signals were not stationary, therefore, the time–frequency analysis was critical in detecting movement, such as a fall. The article used real fall data to demonstrate the efficacy of preexisting models. The initial fall data also aided in revealing some of the difficulties encountered by technology improvements for fall detection.

Ma et al. [54] proposed MHT-PCD-Speed, a revolutionary model-free detection-based tracking technique for identifying and following moving objects in Doppler LiDAR scans. According to the findings, using Doppler radar images can improve tracking reliability and raise the precision of dynamic state estimates.

In [55] Han et al. were using shallow (two and six layers) convolutional neural networks (CNNs) to classify objects based on their radar returns. Authors performed their analysis using around 4000 samples of signals reflected from unmanned aerial vehicles (UAVs), people, cars, and other objects and achieved a modest total classification accuracy of around 0.48 using an augmented version of their dataset.

In yet another example [56] of using CNN architecture for the radar target classification, Stadelmayer et al. proposed constrained CCNs. Parameters of convolutional filters used in the first layer of such networks were learned during the process of training. Reported accuracy of the classification of different human activities (walking, idle, arm movements, etc.) in a controlled experimental environment were above 0.99 which is above other state-of-the-art methods mentioned in the publication.

Wan et al. in [57] solved the plane classification and outlier rejection problems using high-resolution radar (HRR) data and CNN architecture consisting of the classification part and the decoder part (for the target rejection). The authors were able to show the classification accuracy dependencies on the network architecture, the amount of training data, and hyper-parameters, but in all the cases it was well above 0.9. The number of samples used during the training was of the order of 100,000 which is much more than in other discussed publications.

Dadon et al. [58] presented a deep-learning-based technique for classifying ground-moving radar objects. The proposed technique learns the micro-Doppler signatures of radar objects in the 2D radar echoes domain. This study demonstrated that a CNN model can do well in classification. It also demonstrated that efficient data augmentation and regularization increase classification performance and decrease overfitting.

Tiwari et al. [59] developed a unique concatenated CNN model that takes the geolocation type and the radar signal data as input and conducts a binary classification to identify animals and persons. The suggested model has an AUC of 99%.

A common step in the data processing in the aforementioned publications is a representation of the radar data as a spectrogram over a slow time which we also use in our approach.

## 3. Methods

### 3.1. Problem Definition

This section describes the radar target classification problem. Moving targets lit by a radar system exhibit frequency modulation as a result of the time-varying delay between the target and the sensor. As follows from the Doppler effect, the major bulk translation of the object toward or away from the sensor causes a Doppler shift of the echo. The target velocities are estimated by:

(1) $$f_{d}\approx 2\nu \frac{f_{t}}{c}$$

where $f_{d}$ is Doppler frequency, $f_{t}$ is the carrier frequency, ν is target radial velocity, and *c* is the speed of light.

The target, on the other hand, may have sections with extra motions. These can add frequency modulations around the main Doppler shift, i.e., micro-Doppler modulations. Chen [60,61] simulated radar micro-Doppler signatures for a variety of objects, including revolving cylinders, vibrating scatterers, and people targets. The scientists also demonstrated that time–frequency analysis of the received signal is a viable method for extracting the micro-Doppler signature, yielding additional information on the target that can be utilized for classification and recognition. Micro-Doppler may be thought of as a unique signature of the target that gives extra information about the target in addition to current methods for target recognition.

### 3.2. MAFAT Radar Challenge

**Figure 1:**
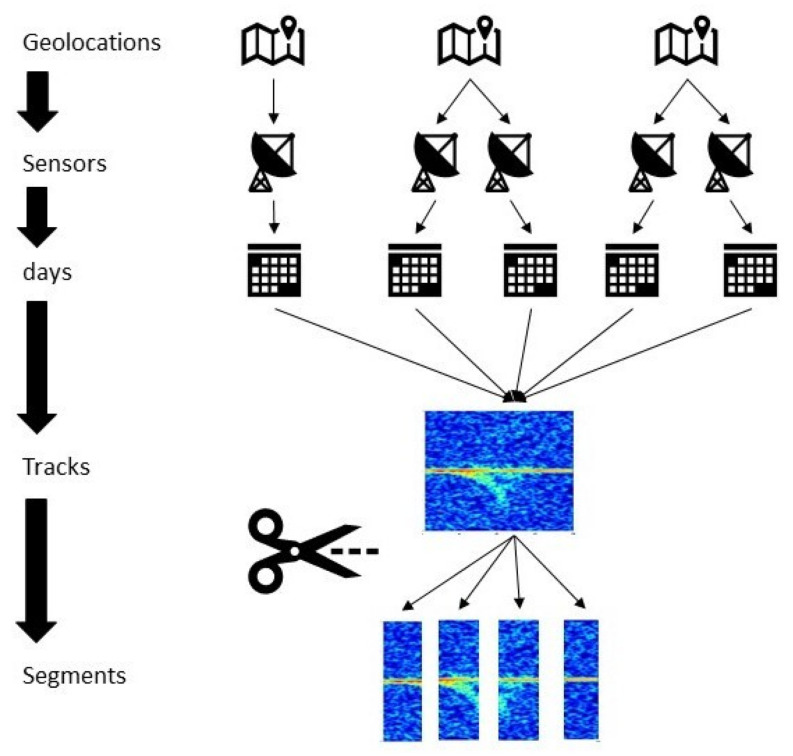
Explanation of MAFAT data.

The goal of the MAFAT radar challenge participants is to classify segments of human or animal radar tracks using an I/Q signal matrix as input (Figure 1). The proposed task is a binary classification task; tracked objects are people or animals. The data is real data collected from different geographical locations, with different time, sensors and quality (i.e., signal to noise ratio (SNR)).

The competition is divided into two parts with different conditions: a public phase with an unlimited number of applications, evaluated on a subset of the public test suite; a private stage where teams are limited to two entries, and where models are evaluated on completely new and unseen data.

The goal of the competition is to classify radar segment data as humans or animals, using ROC AUC as the metric.

#### 3.2.1. Data

The dataset consists of signals recorded by ground Doppler-pulse radar. Each radar “stares” at a fixed, wide area of interest. Whenever an animal or a human moves within the radar’s covered area, it is detected and tracked. The dataset contains records of those tracks. The tracks in the dataset are split into 32 time-unit segments. Each record in the dataset represents a single segment. The dataset is split to training and test sets; the training set contains the actual labels (humans or animals).

A segment consists of a matrix with I/Q values and metadata. The matrix of each segment has a size of 32 × 128. The *X*-axis represents the pulse transmission time, also known as “slow-time”. The *Y*-axis represents the reception time of signals with respect to pulse transmission time divided into 128 equal sized bins, also known as “fast-time”. The *Y*-axis is usually referred to as “range” or “velocity” as wave propagation depends on the speed of light. For example, for pulse repetition interval (PRI) of 128 ms, each *Y*-axis is a bin of 1 ms. For pulse sent in t(n) and a signal received in t(n+m), where 0 < m <= 128, the signal is set in the “m” bin of pulse n (the numbers are not the real numbers and are given only for the sake of the example).

The radar’s raw, original received signal is a wave defined by amplitude, frequency, and phase. Frequency and phase are treated as a single-phase parameter. Amplitude and phase are represented in polar coordinates relative to the transmitted burst/wave. Polar coordinate calculations require frequent sine operations, making calculations time-consuming. Therefore, upon reception, the raw data are converted to Cartesian coordinates, i.e., I/Q values. The values in the matrix are complex numbers: I represents the real part, and Q represents the imaginary part.

The I/Q matrices that are supplied to participants have been standardized, but they have not been transformed or processed in any other way. Therefore, the data represent the raw signal. Different preprocessing and transformation methods, such as Fourier transform, can and should be used to model the data and extract meaningful features. For more information, see “Signal Processing” methods or view the links at the bottom for more information.

The metadata of a segment includes track id, location id, location type, day index, sensor id, and the SNR level. The segments were collected from several different geographic locations, and a unique id was given per location. Each location consists of one or more sensors; a sensor belongs to a single location. A unique id was given per sensor. Each sensor has been used in one or more days, and each day is represented by an index. A single track appears in a single location, sensor, and day. The segments were taken from longer tracks, and each track was given a unique id.

The task of classifying signals to humans and animals is difficult, and it is more challenging in short segments and low SNR signals. One way to view the data is to visualize the signals as a spectrogram. A spectrogram is depicted as a heat map with intensity shown by a color palette.

To generate a spectogram, the I/Q matrix was transformed and processed using Hann windowing and FFT (fast Fourier transform) and calculating the median. We then set as the minimum value of the I/Q matrix and at the end pseudo-coloring.

**Figure 2:**
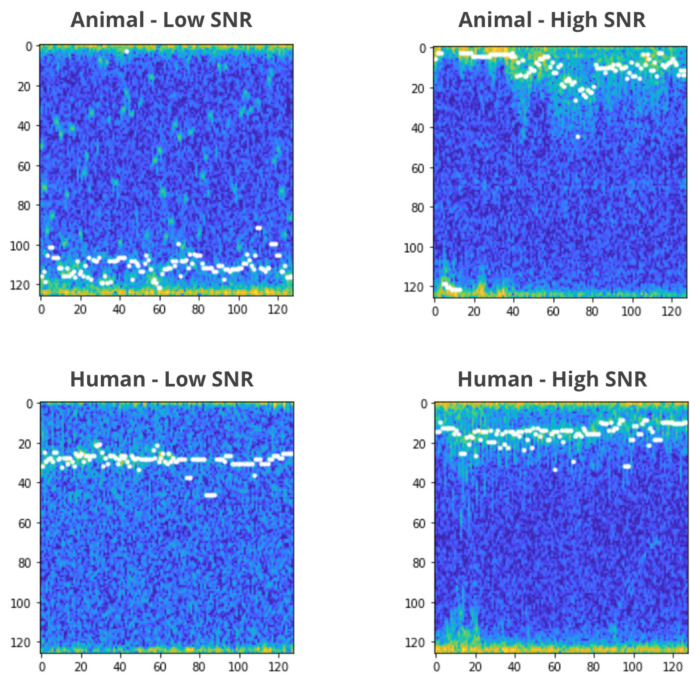
Examples of spectograms.

The images shown in Figure 2 are spectrograms of low and high SNR segments of animals and humans. The white dots are the Doppler burst vector which mark the target’s center-of-mass.

#### 3.2.2. Generalization Considerations

Adjacent segments that can be combined to a whole track can be found in the training and auxiliary datasets but not in the test set. The participants’ goal is to classify every tracked object correctly based on a single segment and not to use the correlation that exists between multiple segments in a track for the classification task. Therefore, most of the records in the test set are single segments that were randomly selected from a full track. In cases where the track was long enough, more than one segment of the same track may be in the test set. Note that they will not be sequential.

The classification should be performed on a single segment level, i.e., the trained models should receive a single segment as input and predict the class of this segment as an output. The class of every segment should be inferred separately based on the features that are extracted only from this specific segment, regardless of any other segment in the test set. The prediction should also be stable, given that the same segment and the same output are expected.

Generalizing to new, unseen, geographic locations such as positioning a radar in a new location changes many things. The terrain, the weather, the objects in the location, and reflections—all these factors may vary from one location to another. The ability to classify a tracked object correctly should be impervious to the changes involved in positioning a radar in new locations. The trained models will be challenged to classify humans or animals on radar tracks that were captured in new location sites, unrepresented in the training set.

The training and test sets contain the following:

- A total of 1510 tracks in the training set;
- A total of 106 segments in the public test set and 6656 segments in the training set;
- In total, there are 566 high SNR tracks and 1144 low SNR tracks in the training set; *200 tracks are high SNR in one part and low SNR in the other;
- In total, there are 2465 high SNR segments and 4191 low SNR segments in the training set;
- Segments are taken from multiple locations. A location is not guaranteed to be a single dataset, but since the goal is to train models that can generalize well to new, unseen, locations—several locations are in the training or the test datasets only;
- It should be mentioned that the data in the training set and in the test set do not necessarily come from the same distribution. Participants are encouraged to split the training set into training and validation sets (via cross-validation or other methods) in such way that the validation set will resemble the test set.

### 3.3. Data Pipeline

The goal of the developed classification module is to classify the already tracked object from the Doppler velocity graphs of this target. The classification module should be able to distinguish between the person and animal while using just Doppler velocities graphs.

Because of their Doppler resemblance, it is difficult to distinguish between people and animals. However, because they are nonrigid entities, changing movements of their sections cause extra modulations in the radar echoes [60]. These micro-Doppler modulations were used for radar target categorization [62]. The categorization of objects using feature extraction by CNNs has received a great deal of attention in the literature [63,64]. It was demonstrated that CNN trained on visual data may exceed human classification capabilities when subjected to visual distortions [63,65,66,67].

Some additional complications that are not present in other datasets collected to test a specific method include the low signal-to-noise ratio, targets that have unexpected/uncontrolled behavior, different sensors used, and other measurement conditions which should not be used while classifying.

### 3.4. Neural Network Design

We investigated the impact of the CNN architecture and data augmentation on the radar target classification performance. Efficient CNN training requires a large, diverse, and well-balanced dataset [68]. The dataset in [49] is small and highly imbalanced.

We studied the effect of the CNN architecture and data augmentation on the classification performance of radar targets. Efficient CNN training necessitates a large, diversified, and well-balanced training dataset, according to [68]. The MAFAT data set [49] is severely unbalanced and tiny. As a result, simple DL algorithms are prone to overfitting. We demonstrated how the proper configuration of well-known regularization approaches may enhance model performance under the ROC-AUC criterion. [69]. We reviewed different CNNs and achieved height performance results in competition by mixing and training different types of CNNs and modifying network layers, selection and training methods, and data balancing techniques to prevent overfitting

**Figure 3:**
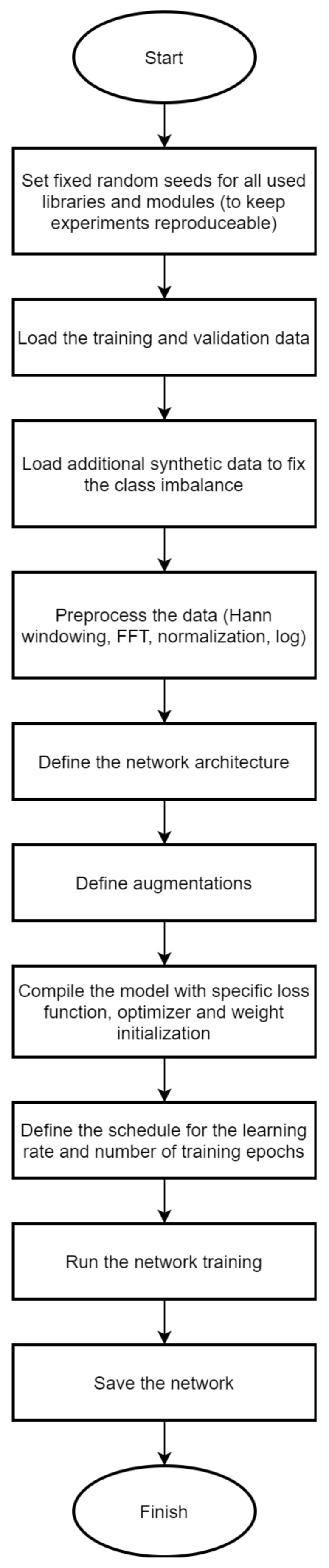
Basic process structure of the radar data classification model training.

Our high-level approach to the competition could be summarized as applying image classification techniques to the preprocessed radar raw data (Figure 3). Some preliminary testing has shown that most simple methods traditionally used for the classification such as logistic regression, linear regression, random forest, and decision tree did not reach the result of the baseline model. The development was focused on data selection, the CNN architecture, and various methods of addressing the overfitting and data leakage which were obvious from the difference between the validation ROC–AUC and the one of the public test set.

**Figure 4:**
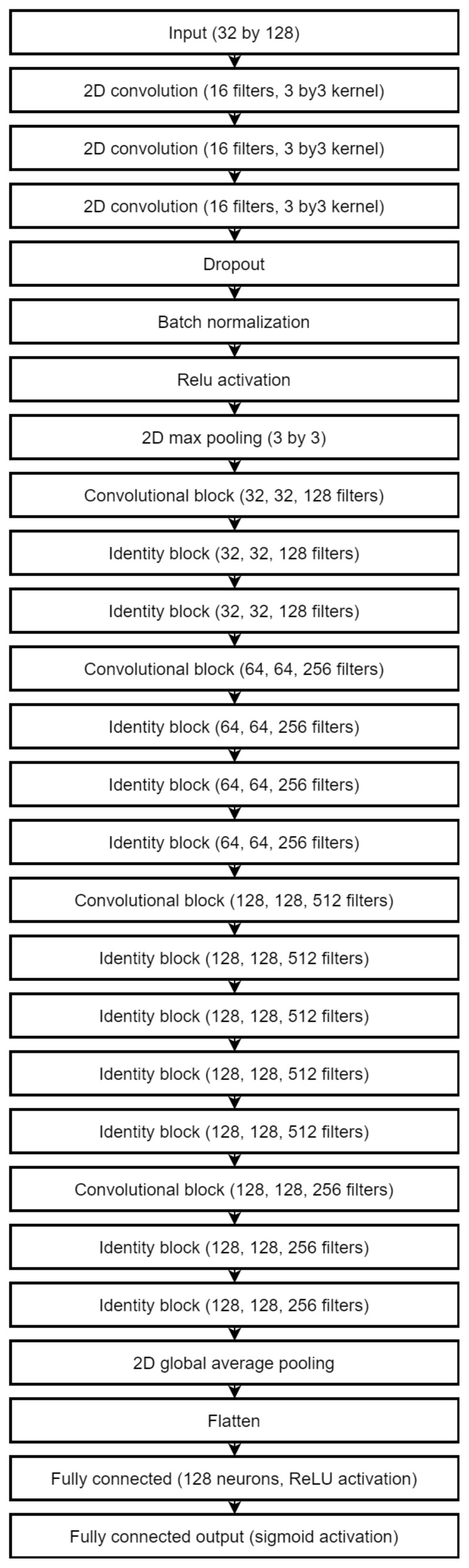
The architecture of the main classification model.

**Figure 5:**
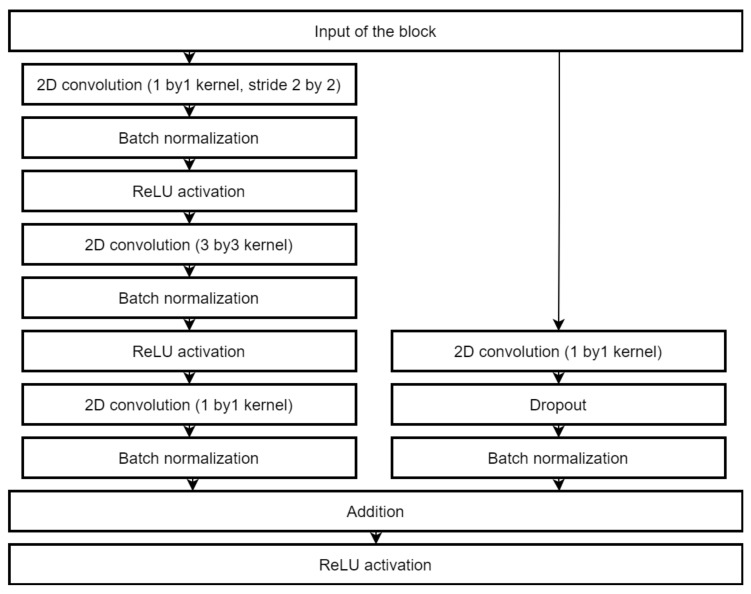
The convolution residual block used in the main classification model.

**Figure 6:**
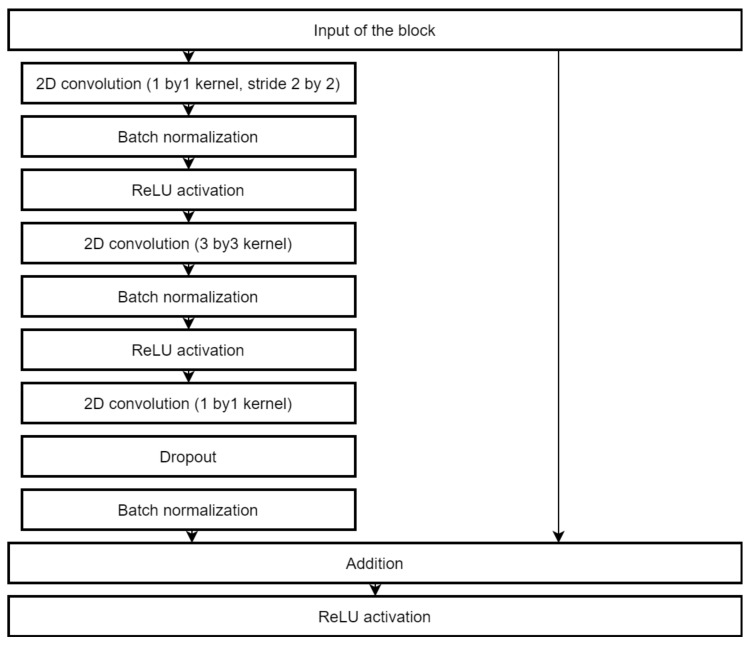
The convolution identity block used in the main classification model.

We applied 82 times to MAFAT [49] for comparing our tests results with the public test set, as it was not available for training or testing, and we only received information about ACU of the applied model. In the final solution, an ensemble of two models was used to improve the accuracy of classification. One architecture is a deep neural network inspired by the ResNet [70]. The complete architecture description is provided in Figure 4, Figure 5 and Figure 6.

**Figure 7:**
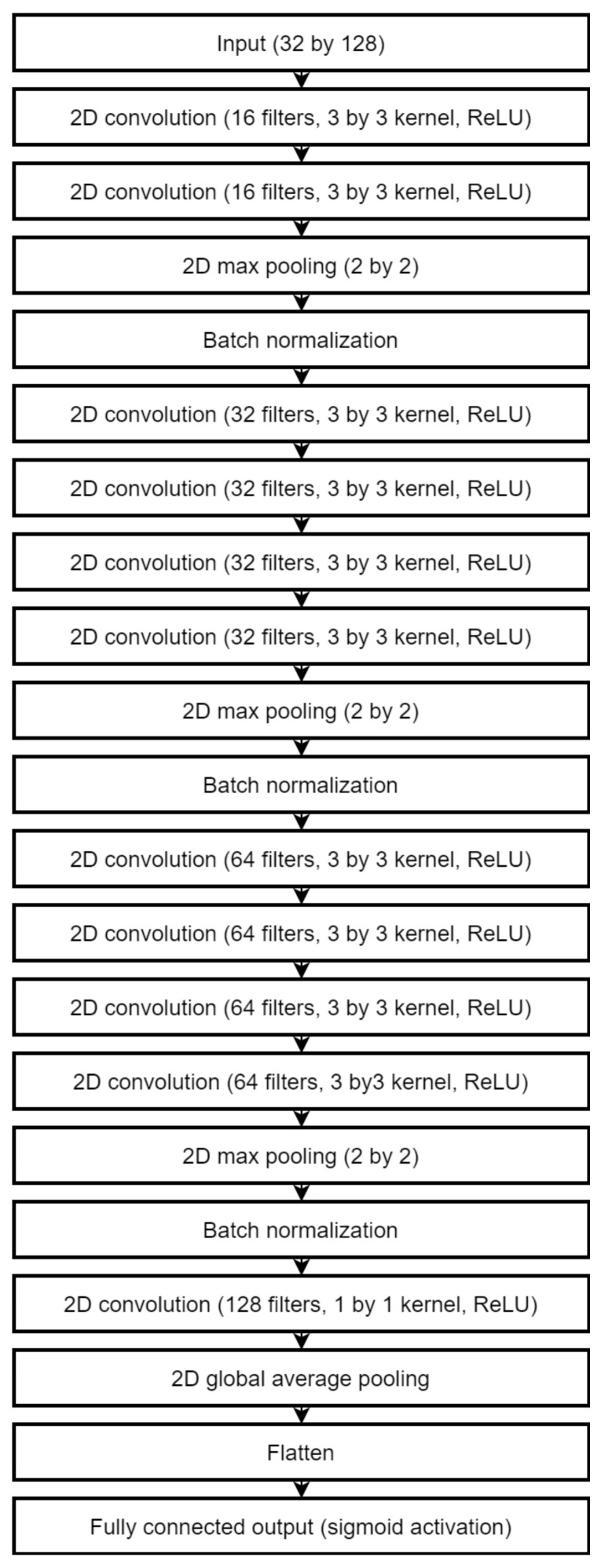
The architecture of the secondary classification model.

A shallower network was used as a secondary classification model and improved the total accuracy and especially the accuracy of true negatives (animal class). This architecture is shown in Figure 7. All convolution layers use ReLU activations. Both networks split into convolution stages which contain two to four convolution layers followed by the batch normalization and max pooling layers. Inside the stage, convolutions use the “same” padding method, which keeps the original size of inputs. Each stage increases the number of filters used but reduces each dimension two times. The weight regularization is used extensively in the secondary network: the L2 norm with weight decay of 0.001 is used in layers before each max pooling and the L1 norm in the final convolutional layer. The L2 norm with the same decay value is used in the fully connected layer of the main network.

## 4. Experiments and Results

### 4.1. Data Set

Data were obtained from different locations and using different radar models during different times of the day. The raw data were I/Q signals grouped into matrices of 32 by 128. The first dimension is the pulse transmission time or the slow time, while the second dimension represents the reception time with respect to the single transmission time or the fast time. The result of the FFT along the second dimension would produce the Doppler velocity graph. No description was given to establish the exact ranges for each of the dimensions, but it was assured that the entire dataset was normalized.

Each 32 × 128 matrix is called a segment. The complete object-tracking event is called a track, and it could take a much longer time than the one during which 32 pulses are transmitted. As a consequence, a single track can have one or more segments that are more or less informative for the task of classification. Although the training dataset contained all the data necessary to construct complete tracks from separate segments, the classification module should classify the object using a single segment without using remaining segments of the same track.

The high imbalance of the data with regard to class of the object was observed. There were many more segments containing animal data than those containing person data (ratio of approximately 5 to 1). Some measurement locations were heavily underrepresented—out of seven different locations, the data were collected in more than half of samples came from the third location. Understanding this, the organizers provided so-called auxiliary experiment data—the data collected at the same locations while performing controlled experiments. In these experiments, much more data of people tracking were obtained. It was not clear at the start, however, if the synthetic data would provide any benefit to the solution.

Finally, some additional data (such as background data for different locations and data with synthetically added noise) were provided.

### 4.2. Data Presprocessing

Some of the most common preprocessing steps, such as loading the data, splitting into training and validation, the FFT, and converting to the logarithmic scale, were provided by the MAFAT radar challenge organizers.

The preprocessing stage was kept from the baseline implementation although different options (such as working with the original I/Q data) were possible. A first Hann windowing function was used to suppress sidelobes resulting after the FFT characteristics. Then, FFT followed and an absolute of the resulting spectrum was taken. Lastly, a logarithm of each value was calculated and result was normalized.

### 4.3. Image Augmentation

Image augmentations is one of the options to reduce overfitting. In the final solution, a very conservative set of augmentations was used:

1. Cyclic width shift of 0.25;
2. Height shift of 0.05;
3. Vertical and horizontal flips of the data.

These augmentations proved to work well while evaluating on the public test set, but the reduced performance on the private test set showed that more image augmentation could be used to reduce the overfitting. The strategy of creating new segments from joined tracks by shifting the sampling of segments from the track in the slow time dimension was considered and tested but did not show meaningful improvement.

At the data exploration step it was quickly observed that adding positive samples from the auxiliary synthetic data not only made the training set more balanced but greatly improved the public test score. Aside from the main training data additional chunk of 7000 segments was loaded from this set in the final solution. Approximately 15,800 total segments were, used for the training 8400 of which were of *person* class.

### 4.4. Model Hyperparameters

The model was compiled with an Adam [71] optimizer which is currently the default choice for the training of convolutional neural networks. Binary cross entropy loss function was used while training.

**Figure 8:**
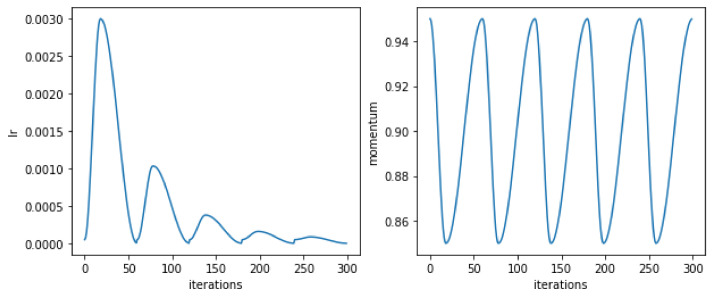
Test run of the learning rate schedule with five cycles produced by a software tool available from [73].

**Figure 9:**
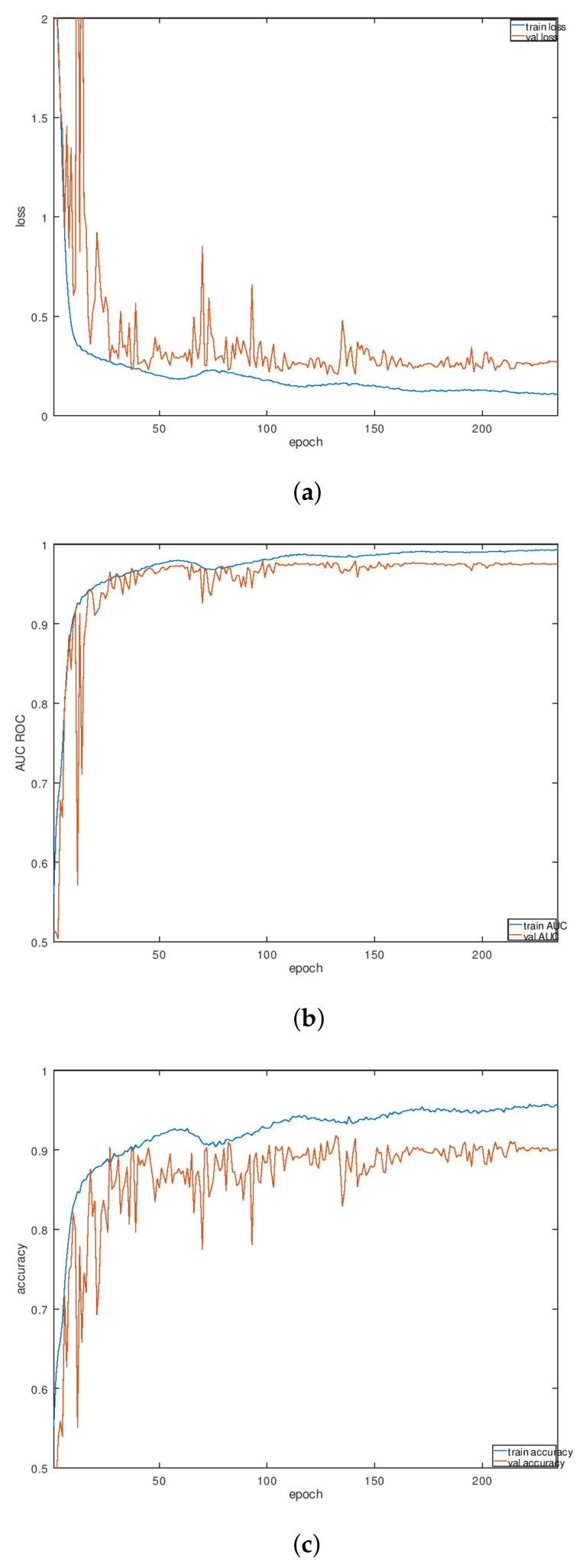
Training performance: (**a**) loss; (**b**) ROC AUC; (**c**) accuracy.

The learning rate was scheduled to increase and drop cyclically (the cyclic learning [72]) which allows it to avoid the weights state staying in a local minima of the multidimensional loss. This proved to be one of the main sources of the score increase. The learning rate started at 0.003 then dropped to 0.00005 at the end of each cycle. CLR implementation for Keras was taken from [73]. The learning process consisting of two to five cycles was tested with the duration of a single cycle between 20 and 45 epochs. A typical learning rate schedule produced by the library is shown in Figure 8. Some typical examples of various metrics obtained in the process of K-fold validation are shown in Figure 9a–c.

### 4.5. Neural Network Training

The splitting into training and validation sets proved to be one of the biggest challenges. The baseline splitting was defined as using three first segments of tracks measured in locations different than those of the training set. The validation set would contain similar numbers of positive and negative examples. After few submissions, it became clear that this validation set does not correlate to the public test—improving ROC AUC from 0.94 to 0.98 would not improve the public test score. To minimize overfitting for the public test, we had to switch to the K-fold validation at some stage of the method development. Special care was taken to avoid the obvious source of the data leakage while evaluating—no two segments of the same track could be used in different folds (Table 1).

The important aspect of the training procedure is that after each cycle, network weights are saved. In the competition, this allowed us to improve the final result by ensembling the same network architecture using different sets (obtained at the end of each cycle) of weights. Although this is impractical while deploying any real-world system, the knowledge distillation could have been implemented to reduce the memory footprint and the processing cost while keeping relatively good performing weights.

## 5. Discussion and Conclusions

This study presented a CNN-based technique for exploiting micro-Doppler signals to classify people and animals using radar. Encouraging results were achieved during the competition—AUC of the ROC around 0.95 on the public test set and above 0.85 on the final (private) test set. Problems of an unbalanced dataset and out-of-sample testing data were the main hurdles while optimizing our processing pipeline. Final results demonstrated a missed opportunity of improving the generalization ability of our networks—an obvious example of overfitting to the public test set was observed. On the other hand, all techniques we employed were a focused effort in this regard, and only the lack of the clear improvement criteria stopped us from achieving even better results.

The proposed DNN architecture, in contrast to more common shallow CNN architectures, is one of the first attempts to use such an approach in the domain of radar data. The usage of the synthetic radar data, which greatly improved the final result, is the other novel aspect of our work. Our solution in the competition could be used as a building block to future implementation of radar classification based on CNN.

During evaluation, we noticed that different network structures perform differently on animal and human recognition, and that is the main reason to use two networks for recognition of humans and animals. Combining the results helps to prevent fouls defections, where there is no target in image, but the network still recognizes one.

Future work is required to optimize the implementation to be able to run calcification in real-time directly since it now requires high resource usage. Exploring competitors, work [58,74] will also improve the solution.

Alternatively, the knowledge distillation technique would be a promising way to reduce the amount of processing and improve the decision latency. Another possible way of improvement is based on the fact that each foster network predicts another object, and if we add more information (data), it will be possible to perform transfer learning and strengthen the accuracy of the model to provide the an option to build an independent system that works in parallel (using Kubernetes).

## Figures and Tables

**Table 1:** Experimental results with different K-folds.

K-Fold Experiments Results			
**K-Fold**	**7**	**5**	**10**
Accuracy	94.035 (±0.5777)	89.708 (±0.5129)	91.855 (±3.0449)
ROC AUC	98.347 (±0.2502)	97.181 (±0.1869)	98.510 (±0.6938)
Loss	0.1776	0.3074	0.2828

## Data Availability

The MAFAT Challenge dataset is available at https://competitions.codalab.org/competitions/25389#learn_the_details-data (accessed on 16 January 2022).
